# Care dependency in geriatric patients undergoing surgical interventions: analysis of contributing factors

**DOI:** 10.1590/1806-9282.20250649

**Published:** 2025-12-05

**Authors:** Demet Fidan, Hande Cengiz Açıl

**Affiliations:** 1 Sakarya University Training and Research Hospital – Sakarya, Turkey.; 2 Sakarya University, Faculty of Health Sciences – Sakarya, Turkey.

**Keywords:** Aged, Dependency, Psychological, Patient care, Surgery

## Abstract

**OBJECTIVE:**

The aim of this study was to determine the care dependency levels of geriatric patients undergoing surgical interventions.

**METHODS:**

The study included patients aged 65 years and older who underwent surgery in the surgical clinics of a research and training hospital. The sample consisted of 150 patients (75 males and 75 females). Data were collected using a Personal Information Form and the Care Dependency Scale during the postoperative period.

**RESULTS:**

The majority of the patients (70.7%) were aged 65–74 years, while 21.3% were aged 75–84 years, and 8% were aged 85 years or older. The mean age was 72.26±7.17 years, and the mean body mass index was 27.35±5.86 kg/m^2^. The average hospital stay was 13.71±18.16 days. Chronic diseases were present in 86% of the patients, with hypertension (78.7%) being the most common. The mean Care Dependency Scale score was 56.02±16.98. Care dependency levels were not significantly affected by alcohol use, chronic disease status, surgical history, body mass index, or hospitalization duration. However, care dependency levels varied significantly by age, sex, education level, marital status, employment status, and smoking habits.

**CONCLUSION:**

An association was observed between increased age and higher care dependency levels. Additionally, female sex, lower education levels, being single, and hospitalization in neurosurgery or orthopedic clinics were associated with higher care dependency. Age-related biopsychosocial changes affect care dependency before and after surgery. Therefore, healthcare professionals—especially nurses—should adopt a holistic approach in providing geriatric care.

## INTRODUCTION

As the number of illnesses and injuries increases, so do care requirements and hospitalizations. Chronic diseases, aging, emotional losses, and changes in physical and psychological health can lead to greater dependency on others for meeting daily needs. Hospitalized patients require careful assessment of potential changes and developments related to their care and treatment to determine their level of independence or need for assistance^1–3^.

Care dependency is defined as a patient's need for professional support, a decline in their ability to perform self-care, and their requirement for a specific level of care based on their dependency status. In care services designed according to dependency levels, the primary goal is to help patients regain independence and return to an active life. For individuals at risk of losing self-care abilities due to health deterioration, care dependency is a fundamental ethical responsibility for nurses^3^. Understanding the independence–dependence status of patients plays a crucial role in shaping the care plan, as dependent individuals require more extensive nursing care compared to independent ones. Their expectations from nursing care also differ accordingly^4^.

Surgical risk stratification remains a critical component in optimizing outcomes, particularly among geriatric populations. Foundational frameworks such as Fried's frailty criteria and the American College of Surgeons National Surgical Quality Improvement Program (ACS NSQIP) guidelines provide essential tools for preoperative assessment. Furthermore, the COVID-19 pandemic has underscored the importance of considering both physical and psychological factors influencing surgical patients. Galhardo et al. conducted a cross-sectional study examining self-reported pain associated with temporomandibular disorders (TMDs) alongside emotional states among medical faculty and students, revealing that heightened levels of stress and anxiety significantly exacerbate pain symptoms^5^. Complementing this, Soares-Júnior et al. provided a comprehensive review focusing on patient safety in gynecological surgery during the pandemic, emphasizing infection control measures and preventive strategies to maintain surgical care quality. Together, these studies highlight the necessity of a multidisciplinary approach to surgical risk management that integrates psychosocial considerations and rigorous safety protocols^6^.

With increasing life expectancy and a growing elderly population, the number of geriatric surgical interventions is expected to rise. This study aims to assess the care dependency status of geriatric surgical patients and examine how their sociodemographic characteristics and medical histories impact their postoperative dependency levels. Ensuring that individuals regain independence and resume active lives is essential^2,7^. In geriatric patients, care dependency can be influenced by chronic diseases, medication use, obesity, stress, immune system weakening, reduced mobility, muscle and bone deterioration, decreased flexibility, urinary and fecal incontinence, and poor sleep quality. In geriatric surgical patients, dependency levels vary based on surgery type, sex, age, social relationships, medication use, past surgeries, and personality traits^8^. To provide a solid theoretical foundation for this study, Dorothea Orem's Self-Care Deficit Nursing Theory has been adopted. This well-established theory emphasizes that an individual's ability to perform self-care activities is critical for maintaining health, and nursing care becomes essential when deficits in self-care exist^9^. Since care dependency directly reflects limitations in self-care capacity, Orem's theory offers a relevant and robust framework to better understand the needs of geriatric surgical patients. Integrating this framework strengthens the conceptual basis of the present research.

## METHODS

Following ethics committee approval and institutional permission, this study was conducted in surgical clinics between July 1, 2022, and January 1, 2023. Patients were informed about the study, and those who agreed to participate provided written informed consent. The sample consisted of 150 patients (75 males and 75 females) who met the inclusion criteria. The power analysis was conducted using Cohen's f=0.25, which corresponds to a medium effect size commonly accepted in geriatric care studies. With an alpha level of 0.05 and a desired statistical power of 0.80, the minimum required sample size was calculated as 128 participants. Since the actual sample size of the study was 150, it was determined that the study is adequately powered.

Inclusion criteria included age ≥65 years, postoperative status, Turkish language proficiency, full orientation, and no auditory or speech impairments.

Participants were excluded from the study if they met any of the following criteria: being under 65 years of age, not in the postoperative period, having no history of surgical intervention, inability to communicate in Turkish, non-cooperative consciousness, presence of hearing or speech impairments, or refusal to participate. Prior to data collection, ethical approval was obtained from the Sakarya University Faculty of Medicine Clinical Research Ethics Committee (Ethics Approval Number: E-71522473-050.01.04-146243-185). Additionally, institutional permissions were secured from the hospital where the study was conducted.

### Data collection instruments

Data were collected using a Personal Information Form and the Care Dependency Scale (CDS).

Personal Information Form: Developed by researchers based on literature, it included questions on age, sex, education, marital status, clinic of admission, hospitalization duration, chronic diseases, smoking/alcohol habits, prior surgeries, and presence/duration of a companion.

CDS: Developed by Dijkstra based on Virginia Henderson's Need Theory, CDS assesses care dependency across 17 items on a 5-point Likert scale (1=completely dependent, 5=almost independent; total score: 17–85)^10^. The Turkish validity-reliability study was conducted by Yönt et al., and permission for use was obtained via e-mail^11^.

Although the CDS was originally developed and validated for use in long-term care settings, it has also been applied in acute hospital environments, including among postoperative and surgical patients^12,13^. Due to its multidimensional structure—encompassing physical, psychological, and social aspects of care—the CDS is considered suitable for evaluating care needs during hospitalization. Its ease of administration and established construct validity support its use in a variety of clinical contexts^14^. Based on these characteristics, the CDS was deemed an appropriate tool for assessing care dependency in the current study population of geriatric surgical patients.

### Statistical analysis

Data were analyzed using IBM SPSS Statistics 26. Descriptive statistics included frequencies, percentages, means, standard deviations, and ranges. Independent-samples t-tests assessed differences between two groups, while one-way analysis of variance (ANOVA) compared multiple groups. Pearson's correlation analysis examined relationships between numerical variables, and Cronbach's alpha tested CDS reliability. Statistical significance was set at p<0.05.

## RESULTS

The patients’ mean age was 72.26±7.17 years, with a mean BMI of 27.35±5.86 kg/m^2^. Notably, 50% of the patients were female, 62% had a primary school education, 65.3% were married, 53.3% were working, 36.7% were smokers, and 9.3% consumed alcohol. The average hospital stay was 13.71±18.16 days. Regarding hospital admission, 37.8% were in general surgery, 26.4% in orthopedics, and 17.6% in gastrointestinal surgical oncology. About 86% had chronic diseases, with the most common being hypertension (78.7%), diabetes (42.5%), and cardiovascular diseases (33.1%). Approximately 82.7% had previous surgeries, and all patients had a companion throughout their stay. The mean CDS score was 56.02±16.98. The Cronbach's alpha coefficient for CDS was above 0.70, indicating good internal consistency.

A significant relationship was found between age and CDS scores (p<0.05). Dependency levels were higher in patients aged 85 years and older compared to those aged 75–84 years and higher in those aged 75–84 years compared to those aged 65–74 years, indicating increased care dependency with age. While alcohol consumption had no significant effect on CDS scores (p>0.05), scores significantly varied based on sex, education, marital status, working status, and smoking status (p<0.05). Male patients, those with university degrees, married patients, employed patients, and non-smokers had significantly lower care dependency, indicating greater independence in their care needs (Table 1).

**Table 1:** Relationships between the Care Dependency Scale Scores and sociodemographic characteristics of the patients.

		Care dependency scale		Test	p
		Mean	SD		
Age (years)	65–74	60.08^a^	16.01	13.230^F^	**0.000** *
	75–84	48.53^b^	15.50		
	85 and above	40.17^b^	13.62		
Gender	Female	50.65	16.37	-4.068^t^	**0.000** *
	Male	61.39	15.95		
Education level	Illiterate	49.34^b^	18.70	2.915^F^	**0.023** *
	Primary school	57.32	16.01		
	Middle school	52.11	14.64		
	High school	54.80	16.19		
	University	69.33^a^	17.01		
Marital status	Married	59.39	16.14	3.455^t^	**0.001** *
	Single	49.67	16.85		
Working status	Not working	50.04	16.58	-4.258^t^	**0.000** *
	Working	61.25	15.63		
Smokers	Yes	63.16	14.87	4.126^t^	**0.000** *
	No	51.88	16.82		
Alcohol	Yes	59.43	17.79	0.788^t^	0.432
	No	55.67	16.92		

a,b Superscripts (a, b) indicate statistically significant differences between groups based on post-hoc comparisons (p<0.05).

F One-way ANOVA test,

t Independent sample t-test.

* p<0.05.

Statistically significant values are denoted in bold. SD: standard deviation.

There was no significant difference in CDS scores based on chronic diseases or a history of surgery (p>0.05). However, CDS scores were significantly associated with the clinic to which patients were admitted (p<0.05). Patients in the gastrointestinal surgical oncology and thoracic surgery clinics had higher CDS scores, indicating greater care dependency, compared to those in other clinics. Additionally, patients in the general surgery clinic were more independent than those in the orthopedics and neurosurgery clinics (Table 2). A significant negative relationship was found between age and CDS scores (p<0.05), meaning care dependency increased with age. No significant relationship was observed between CDS scores and BMI or hospitalization duration.

**Table 2:** Relationships between the Care Dependency Scale Scores and medical characteristics of the patients.

		Care Dependency Scale		Test	p
		Mean	SD		
Clinic	General surgery	49.73^c^	12.14	8.711^F^	**0.000** *
	Orthopedics	67.38^a^	13.80		
	Gastro-oncology	56.55^b^	18.56		
	Thoracic surgery	62.83^a^	9.26		
	Brain surgery	46.31^c^	14.52		
Chronic disease	Yes	55.48	16.65	-0.964^t^	0.337
	No	59.33	18.99		
Surgical history	No	56.35	16.75	0.107^t^	0.915
	Yes	55.95	17.10		

a,b,c Superscripts (a, b, c) indicate statistically significant differences between groups based on post-hoc comparisons (p<0.05).

F One-way ANOVA test,

t Independent sample t-test.

* p<0.05.

Statistically significant values are denoted in bold. SD: standard deviation.

In the analysis of factors affecting care dependency, age, working status, and clinic of admission were significant predictors (p<0.05). Patients aged 75–84 years had CDS scores 7.646 units higher than those aged 65–74 years, while patients aged 85 years or older had scores 17.948 units lower than those aged 66–74 years. Patients who were working had CDS scores 6.840 units higher than those who were not. Patients in gastrointestinal surgical oncology, general surgery, and thoracic surgery clinics had higher CDS scores compared to those in orthopedics (16.146, 8.417, and 10.480 units higher, respectively). Overall, older age and admission to orthopedic clinics were associated with higher care dependency, while employment status was associated with lower dependency (Table 3).

**Table 3:** Factors affecting care dependency.

	Unstandardized coefficient		Standardized coefficient	t	p	95.0%CI		VIF
	B	Std. Hata	Beta			Lower limit	Upper limit	
Constant	41.467	4.136		10.026	0.000	33.291	49.644	
75–84/65–74 years old	-7.646	2.945	-0.185	-2.596	0.010	-13.468	-1.823	1.089
85 years and above/65–74 years old	-17.948	4.435	-0.288	-4.047	0.000	-26.714	-9.182	1.082
Working status (no/yes)	6.840	2.466	0.202	2.774	0.006	1.966	11,714	1.131
Brain surgery/orthopedics	-0.921	4.907	-0.014	-0.188	0.851	-10.622	8.779	1.224
Gastro-oncology/orthopedics	16.146	3.747	0.361	4.309	0.000	8.739	23.553	1.504
General surgery/orthopedics	8.417	3.075	0.241	2.738	0.007	2.339	14.496	1.654
Thoracic surgery/orthopedics	10.480	4.245	0.201	2.469	0.015	2.089	18.871	1.423

F: 10.312, p: 0.000, R^2^: 0.337. CI: confidence interval. B: unstandardized regression coefficients. The t-value measures the size of the difference relative to the variation in your sample data.

## DISCUSSION

This study found that care dependency increased with age, a result supported by several studies in the literature^7,15–21^. Physiological, psychological, and biological changes due to aging contribute to this increase in care dependency. Similar findings have been reported in studies involving hemodialysis patients^22^ and laparoscopic abdominal surgery patients^23^, which showed that aging correlates with higher dependency levels. Aging is associated with a lower quality of life, decreased self-efficacy in self-care, and increased dependency^20^. Female patients in this study were found to be more dependent on others than males, a finding consistent with Caljouw et al.^24^ and Pekince and Aslan^16^, though some studies found no significant relationship between sex and care dependency^4,25,26^. This may be due to females having a more emotional response to the challenges of surgery in the postoperative period. Education level was also found to influence care dependency, with lower education levels associated with higher dependency^4,17,19^. Educated patients tend to adapt better to the postoperative period and maintain higher levels of independence due to better self-care knowledge^27^.

In this study, single or widowed patients were found to be more dependent than married patients. Pekince et al. also reported a significant relationship between marital status and care dependency^16^. Similarly, Enkvist et al. found that marital status significantly affected quality of life^28^. Married patients may have received psychological support from spouses, aiding them in coping with the challenges of aging and hospitalization, which could explain their lower care dependency. However, no significant relationship between marital status and care dependency was found in some other studies^25^.

Working patients were found to be more independent than non-working patients, aligning with Güler et al., who found higher CDS scores in non-working hemodialysis patients^21^. In contrast, Türk and Üstün found lower care dependency among non-working individuals^15^. Working patients may have maintained a higher sense of self-efficacy, which helped them be more independent.

Smokers in this study may have had higher care dependency levels due to the negative impact of smoking on health and the increased prevalence of chronic diseases. Additionally, patients’ care dependency varied based on their clinic of admission. Patients in neurosurgery and orthopedics clinics showed higher dependency levels compared to those in other clinics, a finding consistent with studies on care dependency in various medical specialties^2,4,29^. This could be related to the immobilization following neurosurgery and orthopedic procedures.

While hospitalization duration did not significantly affect care dependency levels in this study, Baksi and Genç found a positive relationship between longer hospital stays and increased dependency^18^. The lack of a significant association in this study may be due to patients focusing on their disease and postoperative complications, with continuous companionship potentially mitigating the effects of extended hospital stays.

A potential confounding factor in this study is the presence of companions during hospitalization for all patients. The assistance provided by companions might artificially lower measured care dependency scores by compensating for patients’ functional limitations. This "companion bias" could mask the true level of dependency, potentially underestimating the care needs of geriatric surgical patients. Future studies should consider stratifying analyses by companion presence or control for this variable to better isolate patients’ intrinsic care dependency. Acknowledging this limitation is important for interpreting the findings and improving external validity.

A potential limitation of this study is that the CDS was originally designed for use in long-term care settings, which may affect its sensitivity in detecting short-term functional changes following surgery. While previous research has demonstrated the feasibility of using the CDS in acute and hospital-based populations, there are limited studies evaluating performance in surgical contexts^13,14^. This limitation should be considered when interpreting our results, as the tool may not fully capture the fluctuating nature of care dependency during the immediate postoperative period. Nevertheless, the CDS offers a structured and comprehensive framework for assessing nursing care needs and remains a clinically useful instrument in geriatric inpatient settings.

One important limitation of this study is the exclusion of older adults with cognitive or auditory impairments—conditions frequently encountered in geriatric populations. This exclusion may have introduced selection bias, resulting in an overrepresentation of functionally independent and healthier elderly individuals. Consequently, the generalizability of our findings to more vulnerable subgroups, such as patients with dementia or sensory deficits, is limited. Previous studies have similarly noted that individuals with cognitive impairment are often underrepresented in clinical research due to perceived challenges in informed consent and data reliability^30,31^. To ensure more inclusive and representative results in future studies, modified assessment tools, caregiver input, or proxy reporting may be necessary to capture the full spectrum of care dependency in geriatric surgical populations.

### Strengths and limitations

#### Strengths

Novel focus: This study offers a rare and valuable examination of care dependency among geriatric surgical patients.

Gender balance: Equal representation of male and female participants enhances the generalizability of the results.

Clinical relevance: The study identifies high-risk subgroups, such as orthopedic and neurosurgery patients, informing targeted clinical management.

#### Limitations

The study's sample size was limited by the high number of ambulatory surgeries during the research period.

One key limitation of this study is the exclusion of patients with cognitive and auditory impairments, who are commonly encountered in the geriatric population. Although this criterion was necessary to ensure accurate self-reporting during the application of the CDS, it may have led to the overrepresentation of healthier and more cognitively intact individuals. As a result, the generalizability of the findings is limited, and the actual care dependency levels among more vulnerable geriatric patients may have been underestimated.

Some critical confounding variables, such as pre-operative functional status, frailty scores, type of anesthesia, and surgical complexity, were not included in the analysis despite their known influence on postoperative dependency. Due to data limitations, these covariates could not be controlled for in the multivariate models. This constitutes an important limitation of the study and should be considered when interpreting the results. Future research is recommended to incorporate these factors to better understand their impact on patient outcomes.

## CONCLUSION

This study highlighted that factors such as age, sex, education level, marital status, working status, and smoking significantly influenced the care dependency levels of geriatric surgical patients. However, alcohol consumption, chronic diseases, BMI, hospitalization duration, and previous surgeries did not show a significant impact on care dependency. Older patients undergoing surgery were observed to have higher care dependency levels compared to younger patients. To reduce patient anxiety, it is essential to provide clear information and involve both patients and their families in the treatment process. Nurses and healthcare providers should be well trained in addressing the unique physiological and psychological changes of elderly patients to ensure better care outcomes.

## Data Availability

The datasets generated and/or analyzed during the current study are available from the corresponding author upon reasonable request.
